# Copolymeric Micelles of Poly(ε-caprolactone) and Poly(methacrylic acid) as Carriers for the Oral Delivery of Resveratrol

**DOI:** 10.3390/polym15183769

**Published:** 2023-09-14

**Authors:** Katya Kamenova, Lyubomira Radeva, Spiro Konstantinov, Petar D. Petrov, Krassimira Yoncheva

**Affiliations:** 1 Institute of Polymers, Bulgarian Academy of Sciences, Akad. G. Bonchev Str. 103A, 1113 Sofia, Bulgaria; kkamenova@polymer.bas.bg; 2 Faculty of Pharmacy, Medical University of Sofia, 2 Dunav Str., 1000 Sofia, Bulgaria; l.radeva@pharmfac.mu-sofia.bg (L.R.); skonstantinov@pharmfac.mu-sofia.bg (S.K.)

**Keywords:** polymeric micelles, block copolymers, poly(ε-caprolactone), poly(methacrylic acid), resveratrol, anti-inflammatory activity

## Abstract

In this study, we report the development of a micellar system based on a poly(methacrylic acid)-*b*-poly(ε-caprolactone)-*b*-poly(methacrylic acid) triblock copolymer (PMAA_16_-b-PCL_35_-b-PMAA_16_) for the oral delivery of resveratrol. The micellar nanocarriers were designed to comprise a PCL core for solubilizing the poorly water-soluble drug and a hydrated PMAA corona with bioadhesive properties for providing better contact with the gastrointestinal mucosa. The micelles were first formed in an aqueous media via the solvent evaporation method and then loaded with resveratrol (72% encapsulation efficiency). Studies by transmission electron microscopy (TEM) and dynamic and electrophoretic light scattering (DLS and PALS) revealed a spherical shape, nanoscopic size (100 nm) and a negative surface charge (−30 mV) of the nanocarriers. Loading of the drug slightly decreased the hydrodynamic diameter (Dh) and increased the ƺ-potential of micelles. In vitro dissolution tests showed that 80% and 100% of resveratrol were released in 24 h in buffers with pH 1.2 and 6.8, respectively, whereas for the same time, not more than 10% of pure resveratrol was dissolved. A heat-induced albumin denaturation assay demonstrated the advantage of the aqueous micellar formulation of resveratrol, which possessed anti-inflammatory potential as high as that of the pure drug. Further, the micellar resveratrol (5 µM) exerted a strong protective effect and maintained viability of mucosa epithelial HT-29 cells in a co-cultural model, representing the production of inflammatory cytokines. For comparison, the pure resveratrol at the same concentration did not protect the damaged HT-29 cells at all. Thus, the present study revealed that the PMAA-*b*-PCL-*b*-PMAA copolymeric micelles might be considered appropriate nanocarriers for the oral delivery of resveratrol.

## 1. Introduction

In the last two decades, the nanomedicine approach for prophylaxis, diagnosis, and treatment of numerous diseases has received remarkable attention. Exploiting nanocarriers of predetermined properties and functions is proven as an effective strategy for achieving sophisticated delivery of therapeutic agents, thus overcoming the disadvantages encountered in traditional treatments [1,2,3]. These nanosystems are employed as transporting agent for vaccines, drugs, genes, proteins and enzymes, as they have potential to prolong the circulation time in the blood stream and accumulate at the target sites. Numerous nanocarrier-based medical products are undergoing preclinical and clinical trials for different diseases. and some of them were approved by the US Food and Drug Administration (FDA) and the European Medicines Agency (EMA), and reached the global market [4]. Nanocarriers have many advantages such as biocompatibility, biodegradability, small size, nonimmunogenicity, high drug loading capacity, and controllable drug release. Their physicochemical properties such as size, charge, and surface properties can be tuned to provide sufficient in vivo stability and favorable biodistribution, enhanced uptake by the cells, and targeting ability.

The oral administration of drugs is one of the most common and highly preferred route, compared to parenteral, nasal, and topical routes, because it is convenient, economical, non-invasive, painless, and safe for patients [5]. However, most of the existing and newly developed drugs are characterized by poor water solubility, which is a critical determinant of their dissolution rate, and usually leads to low oral absorption and bioavailability, poor pharmacokinetics, and possible irritation. In addition, various drugs can be degraded by the action of enzymes or the acidic environment in the stomach [6]. Micellar nanoparticles, formed by self-assembly of amphiphilic block copolymers with biodegradable and/or biocompatible chains, can be used as carriers for the oral delivery of hydrophobic drugs. Such nanocarriers could increase the solubility of drugs, prevent their degradation, reduce side effects, and improve drug bioavailability [7,8]. Micellar formulations with physically entrapped or covalently linked into the hydrophobic core drugs exhibited sustained drug release profiles, controlled by the erosion and/or dissolution of the encapsulating matrix [9]. Furthermore, the formulation of micelles possessing a bioadhesive shell could provide longer gastrointestinal residence and improvement of interactions with epithelial cells. The structural stability of the micelles is a determining factor for achieving the abovementioned functions. The stability of the micelles depends on various factors such as the nature of the building blocks, hydrophilic/hydrophobic balance, glass transition temperature of the hydrophobic blocks, etc. [10]. Poly(ε-caprolactone) (PCL) is a FDA-approved semi-crystalline biodegradable and biocompatible polyester with a low melting point of around 60 °C and a glass transition temperature of about −60 °C, which makes it suitable for developing nano-based drug delivery systems. Nanocarriers based on PCL have been explored to enhance the therapeutic efficiency of various drugs [11,12]. Peng et al. developed a biodegradable polymeric carrier based on amphiphilic methoxy poly(ethylene glycol)-*b*-poly(ε-caprolactone) block copolymer for oral delivery of the water-insoluble drug capsaicin [13]. The micellar formulation increased capsaicin bioavailability compared to the control formulation.

Poly(methacrylic acid) (PMAA) is an appropriate polymeric carrier for oral drug delivery systems because of its bioadhesive properties that ensure longer residence in gastrointestinal tract and enable the contact with the epithelium. In addition, PMAA is a weak polyelectrolyte (the degree of ionization is governed by the pH and ionic strength of aqueous solution) able to provide pH-sensitive release of the drug [14]. Satturwar et al. [15] developed pH-sensitive micellar nanocarriers from poly(ethylene glycol)-*b*-poly(alkyl(meth)acrylate-co-methacrylic acid) for delivery of the poorly water-soluble compound candesartan cilexetil. They observed substantially increased release rate of the drug from the polymeric micelles when the pH was shifted from 1.2 to 7.2 [15].

Resveratrol (RES) is a polyphenol, more specifically, a hydroxylated derivative of stilbene, which is produced by *Vitaceae* members in a non-specific response to injury or infection [16]. It exists in two isomeric forms (trans- and cis-resveratrol); however, the trans- form is found to possess more pronounced pharmacological effects [17]. The main pharmacological activities of resveratrol are related to its antioxidant, anti-inflammatory, anticancer, antiaging, vasorelaxant, estrogenic, antibacterial, antifungal, antivirus, neuro- and cardioprotective effects [18,19,20,21]. Regarding anti-inflammatory activity, the polyphenol influences various signaling pathways and it is able to regulate inflammation via different mechanisms [22,23,24,25]. Its anti-inflammatory activity is of a particular importance since inflammation is a part of the pathology of a huge number of common diseases such as diabetes, cardiovascular, respiratory and neurological diseases, cancer [26]. Nowadays, the chronic and life-threatening inflammatory gastrointestinal diseases affect a large part of the population [27,28]. Applying resveratrol in therapies of inflammatory gastrointestinal diseases could be a promising new strategy because its antioxidant activity might inhibit inflammation due to a capture of radicals generated during the process. However, the application of resveratrol is restricted since it has extremely low aqueous solubility (50 µg/mL), high lipophilicity (logP = 3.1) [29] and low bioavailability due to an extensive metabolism [30]. Incorporation of resveratrol into nanoparticles, and especially into micelles that possess an optimal size, is a promising approach to dealing with its shortcomings and improving the efficacy of such therapies. For instance, Kamel et al. improved the anti-inflammatory effect of resveratrol on rats with induced arthritis by encapsulating it in poly(lactic acid)-coated Pluronic P188 and P407 micelles [31]. Loading resveratrol into pH-responsive poly(ethylene glycol)-acetal-polycaprolactone-poly(ethylene glycol) micelles, modified with cyclic arginine-glycine-aspartic acid and triphenylphosphine, could ensure better penetration through the blood–brain barrier and enhanced alleviation of oxidative stress and inflammation [32].

The aim of the present work was the elaboration of a novel PMAA-*b*-PCL-*b*-PMAA-based micellar carrier for the oral delivery of a highly hydrophobic drug as resveratrol. The amphiphilic block copolymer, comprising a central PCL hydrophobic block and two outer PMAA hydrophilic blocks, was synthesized via atom transfer radical polymerization (ATRP) of *tert*-butyl methacrylate (*t*-BMA), initiated from a bifunctional PCL macroinitiator, followed by hydrolysis of *t*-BMA groups. Stable micelles were obtained by self-assembly of the copolymer in aqueous media, and then loaded with the hydrophobic polyphenol resveratrol. The main physicochemical characteristics of blank and drug-loaded micelles were determined as well. The potential of the resveratrol-loaded micelles as an oral anti-inflammatory delivery system was explored via two in vitro models, particularly a heat-induced albumin denaturation assay and a cytotoxic damage of colorectal cells (HT-29) via differentiated eosinophilic cells.

## 2. Materials and Methods

### 2.1. Materials

Poly(ε-caprolactone) diol (CAPA^®^ 2402, molar mass 4000 g mol^−1^; Solvay Chemicals Inc., Houston, TX, USA) was precipitated in cold methanol (−30 °C), filtered and dried under vacuum at 40 °C overnight. Tert-butyl methacrylate (tBMA, Sigma-Aldrich, FOT, Sofia, Bulgaria) was passed through column of alumina to remove the inhibitor. Copper (I) bromide (98%, CuBr), pentamethyldiethylenetriamine (99%, PMDETA), α-bromoisobutyryl bromide (98%, BIBB), triethylamine (99.5%, TEA), trifluoroacetic acid (99%, TFA), 1,6-diphenyl-1,3,5-hexatriene (98%, DPH), anisole (99%), dioxane (99%), acetone (99%), trans-resveratrol, bovine serum albumin (fraction V) and phorbol 12-myristate 13-acetate (PMA) were purchased (Sigma-Aldrich, FOT, Sofia, Bulgaria) and used as received. Dichloromethane (99.8%, DCM, Sigma-Aldrich, FOT, Sofia, Bulgaria) and tetrahydrofuran (THF, HPLC grade, Fisher Chemical, Labimex, Sofia, Bulgaria) were stirred overnight in calcium hydride (95%, Sigma-Aldrich, FOT, Sofia, Bulgaria) and distilled before used. The dialysis membrane (standard-grade regenerated cellulose, 10,000 MWCO, Spectrum Labs, San Francisco, CA, USA) was purchased from Fisher Scientific (Labimex, Sofia, Bulgaria). Human colorectal adenocarcinoma (HT-29) and human eosinophilic (EOL-1) cell lines were obtained from the German Collection of Microorganisms and Cell Cultures (DSMZ GmbH, Braunschweig, Germany). Roswell Park Memorial Institute 1640 Medium and 3-(4,5-dimethylthiazol-2-yl)-2,5-diphenyltetrazolium bromide (MTT) were purchased from Sigma-Aldrich (Merck KGaA, Darmstadt, Germany).

### 2.2. Analysis

Nuclear magnetic resonance (^1^H-NMR) spectra were recorded with a Bruker Advance II + 600 spectrometer at room temperature using deuterated chloroform (CDCl_3_) or deuterated dimethyl sulfoxide (DMSO-d_6_) as solvents. The number average molar mass (M_n_) and dispersity index (DI, M_w_/M_n_) were determined by gel permeation chromatography (GPC) with a Shimadzu Nexera HPLC chromatograph, equipped with a degasser, a pump, an autosampler, a RI detector and three PSS SDV columns (5 μm Linear M; 5 μm, 100 Å; and 5 μm, 50 Å), using tetrahydrofuran as the eluent at a flow rate of 1.0 mL/min and a temperature of 40 °C. The concentration of samples was 1 mg/mL and the instrument was calibrated with polystyrene standards.

The hydrodynamic diameter of micelles was measured by using a Zetasizer NanoBrook 90Plus PALS, equipped with a 35 mW red diode laser, (λ = 640 nm) at 37 °C and a scattering angle of 90°. The ƺ-potential was determined from electrophoretic light scattering measurements, conducted on the same apparatus at 37 °C and a scattering angle of 15°, applying the phase-analysis light scattering (PALS) method. The ultraviolet–visible absorption spectra were recorded on a UV-vis spectrophotometer (Thermo Scientific, Waltham, MA, USA) using quartz cells with a path length of 1 cm. The morphology of blank and resveratrol-loaded polymeric micelles was investigated by transmission electron microscopy using HRTEM JEOL JEM-2100 transmission electron microscope operating at 200 kV. A drop of each sample was deposited on a carbon-coated grid and allowed to evaporate prior the analysis.

### 2.3. Synthesis of PMMA-b-PCL-b-PMMA Triblock Copolymer

#### 2.3.1. Synthesis of Br-PCL_35_-Br Macroinitiator

HO-PCL_35_-OH (30.0 g, 3.75 mmol) was dissolved in 30 mL of toluene and dried by azeotropic distillation. In a 100-mL single-neck round-bottom flask equipped with a magnetic stirrer; 2.612 g (10 mmol) of the dry polymer was dissolved in freshly distillated THF. Then, triethylamine (5.16 mL, 37 mmol, 1.8 eq) was added to the solution and the reaction mixture was cooled to 0 °C by dint of an ice bath. 2-bromoisobutyryl bromide (3.71 mL, 30 mmol, 1.8 eq) was added dropwise via syringe then the mixture was stirred at room temperature for 20 h. The formed precipitate of ammonium bromide was removed by filtration and the solution was concentrated by rotary vacuum evaporation. Then, the final product was isolated by precipitation into a 10-fold excess of cold methanol (−30 °C), recovered by filtration and dried under vacuum at 60 °C overnight. Yield 80%. ^1^H-NMR (CDCl_3_, δ ppm): a 4.06–3.9 (m, 2H, -CH_2_-O-), d 2.31 (m, 2H, -C(O)-CH_2_-), e 1.93 (s, 12H, -C-CH_3_ end groups), b 1.65 (m, 4H, -C(O)-CH_2_-CH_2_-CH_2_-CH_2_-), c 1.39–1.35 (m, 2H, -CH_2_-CH_2_-CH_2_-).

#### 2.3.2. Synthesis of PtBMA_16_-*b*-PCL_35_-*b*-PtBMA_16_ Triblock Copolymer

A 100 mL Schlenk flask equipped with a stir bar was dried under vacuum and then charged with Br-PCL_35_-Br (0.5 g, 0.125 mmol, 1 eq.) under argon. The calculated amount of Cu(I)Br (0.035 g, 0.25 mmol, 2 eq.), and anisole (3 mL) were added sequentially. The flask was sealed off with a septum, and the solution was left stirring at ambient temperature under argon flow for 20 min. After that, the deoxygenated initiator PMDETA (0.052 mL, 0.25 mmol, 2 eq.) was added, and the reaction mixture was allowed to stir under argon atmosphere for an additional 20 min to form the Cu complex. The deoxygenated *t*BMA (0.771 mL, 4.475 mmol, 38 eq.) was added and the flask was kept in a thermostatically controlled oil bath at 70 °C for 22 h. Upon completion of the polymerization, the mixture was diluted with anisole and then the solution was passed through a silica column to remove the copper catalyst. The obtained polymer solution was concentrated by vacuum evaporation and then precipitated into cold methanol. The resulting polymer was filtered and recovered by freeze drying. Yield 78%. ^1^H NMR (CDCl_3_, δ ppm): a 4.06–3.9 (m, 2H, -CH_2_-O-), d 2.31 (m, 2H, -C(O)-CH_2_-), b 1.65 (m, 4H, -C(O)-CH_2_-CH_2_-CH_2_-CH_2_-), j 2.07–1.8 (m, 2H, -CH_2_-C(CO)-), h 1.42 (m 9H, CH_3_-(CO)O-), c 1.39–1.35 (m, 2H, -CH_2_-CH_2_-CH_2_-), f 1.14–1.08 (m, 3H, CH_3_-C(CO)-CH_2_-).

#### 2.3.3. Synthesis of Amphiphilic PMMA_16_-*b*-PCL_35_-*b*-PMMA_16_ Triblock Copolymer

In a 100-mL round-bottom flask, fitted with a stir bar, the P*t*MBA_16_-*b*-PCL_35_-*b*-P*t*MBA_16_ copolymer (0.3 g, 0.03 mmol) was dissolved in freshly distilled dichloromethane (3 mL). After that, trifluoroacetic acid (0.482 mL, 6 mmol) was added and the reaction mixture was stirred at room temperature for 48 h. The resulting copolymer was precipitated in cold methanol (−30 °C), filtered, then the obtained precipitate was dissolved in 1,4-dioxane and freeze-dried. Yield 73%. ^1^H NMR (DMSO-d_6_, δ ppm): i 12.4–12.1 (s, 1H, -CO(O)H), a 4.06–3.9 (m, 2H, -CH_2_-O-), d 2.31–2.21 (m, 2H, -C(O)-CH_2_-), b 1.65 (m, 4H, -C(O)-CH_2_-CH_2_-CH_2_-CH_2_-), j 2.07–1.8 (m, 2H, -CH_2_-C(CO)-), c 1.39–1.25 (m, 2H, -CH_2_-CH_2_-CH_2_-), f 1.14–1.08 (m, 3H, CH_3_-C(CO)H-).

### 2.4. Preparation of Micelles and Determination of Critical Micelle Concentration

The polymeric micelles were prepared by the solvent evaporation method. The amphiphilic triblock copolymer (total mass 0.015 g) was dissolved in 5 mL of acetone/THF mixture (1:1), and the solution was added dropwise to 15 mL of deionized water at room temperature under stirring (800 min^−1^). The resulting dispersion was stirred for 30 min and the organic solvent was evaporated using a rotary vacuum evaporator to afford stable aqueous micellar solution with a concentration of 1 gL^−1^.

The critical micelle concentration (CMC) was determined spectrophotometrically by a method employing the hydrophobic dye 1,6-diphenyl-1,3,5-hexatriene (DPH). Aqueous copolymer solutions (2 mL) with different concentrations (between 0.005 and 1 gL^−1^) were prepared and methanol solution of DPH (20 μL, 0.4 mM) was added to each sample. The samples were incubated in a dark place for 16 h at room temperature before recording the UV–vis absorption spectra of DPH in the wavelength range 250–600 nm at 25 °C (Beckman Coulter DU 800 UV–vis spectrometer). The CMC values were determined as the inflection points of the absorbance intensity (at 356 nm) vs. polymer concentration curve.

The resistance of micelles against degradation in an acidic medium was assessed by adding DPH-loaded micelles in a buffer with pH = 1.2. After storage for 3 h, the UV–vis absorption spectrum of DPH was recorded.

### 2.5. Drug Loading

The encapsulation of resveratrol in the prepared micelles was conducted via the solvent evaporation method. First, an ethanol solution of resveratrol (1 mL) was added to 3 mL aqueous dispersion containing the pre-formed micelles. The system was stirred (700 rpm) at room temperature until evaporation of the ethanol. Thereafter, the loaded micelles were filtered (0.22 µm) and the filter was rinsed with 50% ethanol. The concentration of non-loaded resveratrol in the rinsing fraction was measured spectrophotometrically at 306 nm (Thermo Fisher Scientific, Waltham, MA, USA). A standard curve of resveratrol in 50% ethanol at the range of 2–10 µg/mL (r > 0.9996) was used to calculate the concentration. The following equations were used for determination of the encapsulation efficiency (EE) and the loading degree (LD) of resveratrol:

EE = (Total mass of drug − non-encapsulated drug)/Total mass of drug(1)

LD = (Total mass of drug − non-encapsulated drug)/Volume of drug loaded micellar dispersion(2)

### 2.6. In Vitro Drug Release

The in vitro release tests were performed via dialysis method in buffers (pH of 1.2 and 6.8) containing 10% ethanol. An exact volume amount of micellar dispersion (containing 0.192 mg of resveratrol) was placed in a dialysis membrane (10,000 MWCO). Then, the membrane was introduced in 20 mL buffer and gently shaken at 37 °C (IKA Labortechnik HS-B20, Staufen, Germany). A volume of 1.5 mL was taken from the acceptor phases at predetermined time intervals and the amount of released resveratrol was determined UV-spectrophotometrically at 306 nm (Thermo Fisher Scientific, Waltham, MA, USA). Equal amounts of fresh buffers were returned back at each time point in order to maintain sink conditions.

### 2.7. Albumin Denaturation Assay

The albumin denaturation assay was conducted according to previously reported procedures after minor optimization [33,34,35]. Briefly, pure and loaded into the micelles resveratrol (1 mL, 100 µg/mL) were mixed with an aqueous solution of bovine serum albumin (1 mL, 2% wt/wt). Thereafter, the pH of the mixtures was adjusted to 6.3 by adding sufficient amount of 1 M hydrochloric acid. The probes were incubated at 37 °C for 20 min and then heated at 70 °C for 5 min. After, they were left to cool down and the turbidity was measured by UV-spectrophotometry at a wavelength of 660 nm (Thermo Fisher Scientific, Waltham, MA, USA). Similar mixtures were prepared with distilled water (negative control) and diclofenac sodium (DF, 100 µg/mL, positive control). The percentage of inhibition of albumin denaturation was calculated using the following equation:

Percentage inhibition = [(A_negative control_ − A_sample_)/A_negative control_] × 100 (3)

### 2.8. In Vitro Cytoprotective Effect

EOL-1 and HT-29 cell lines were seeded in 24-well plates at a density of 3 × 10^5^ and 2.5 × 10^5^, respectively, and were incubated overnight at standard conditions of 37 °C, 5% CO_2_ and high humidity (Esco CelCulture^®^ CO_2_ Incubator, CCL-170B-8-IVF, Esco Micro Pte. Ltd., Singapore). Thereafter, EOL-1 cells were treated with 10 µg/mL phorbol 12-myristate 13-acetate (PMA), and HT-29 cells were incubated with empty micelles, pure resveratrol and resveratrol encapsulated in micelles at 5 and 10 µM concentrations for 24 h. Then, the PMA-treated leukemic EOL-1 cells, differentiated into functionally active eosinophilic cells, were washed with PBS and admixed with the pre-treated HT-29 cells in order to damage HT-29 epithelial monolayers for 48 h. The non-damaged HT-29 group, incubated only with the fresh medium, was used as a negative control. PMA-damaged HT-29 cells that were not pre-treated with pure and micellar resveratrol were used as a positive control. After, the cells were rinsed with PBS and new medium with MTT solution was added to each well. The cells were incubated for 3 h at 37 °C and then the formazan crystals obtained were dissolved via addition of 5% formic acid in 2-propanol. The absorbance was then measured using a microprocessor-controlled microplate reader (Labexim LMR-1 Labexim, Lengau, Austria) at 550 nm.

### 2.9. Statistical Analysis

The experiments were conducted in triplicate and the results are presented as mean values ± SD. The statistical analyses were performed using GraphPad Prism 8 software (Dotmatics, San Diego, CA, USA). One-way ANOVA test with Dunnett’s post-test was used for comparison of all test groups to controls (diclofenac sodium group in albumin denaturation assay and the damaged with differentiated eosinophilic cells HT-29 cells in cellular model). For comparison between the different groups, *t*-tests with Holm–Sidak correction were applied.

## 3. Results and Discussion

In the present work, an amphiphilic triblock copolymer comprising biocompatible and biodegradable hydrophobic block of poly(ε-caprolactone) and bioadhesive, pH-sensitive hydrophilic blocks of poly(methacrylic acid) (PMAA) was synthesized, aiming to fabricate micellar nanocarriers for the oral delivery of resveratrol. The main advantage of these micelles could be an improvement of biopharmaceutical properties of the extremely hydrophobic resveratrol and possibility for its oral administration as a stable aqueous micellar dispersion.

### 3.1. Synthesis and Characterization of Copolymer

**Figure 1:**
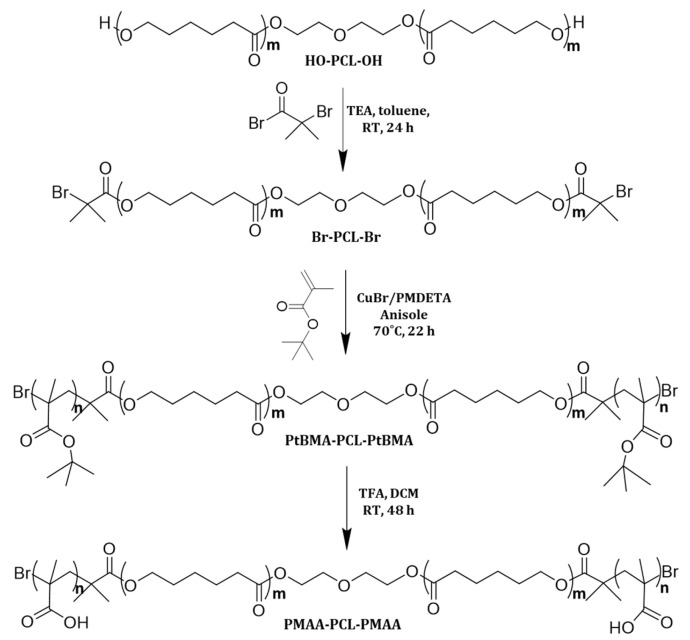
Synthesis of poly(methacrylic acid)-*b*-poly(ε-caprolactone)-*b*-poly(methacrylic acid) copolymer via ATRP of *tert*-butyl methacrylate from PCL-based bifunctional macroinitiator, and subsequent hydrolysis of the poly(t-butyl methacrylate) blocks with trifluoroacetic acid.

Block copolymers containing poly(methacrylic acid) segments belong to the class of polyelectrolyte polymers, which are often selected for elaborating pH-sensitive nanocarriers [14]. Here, the well-defined PMAA-*b*-PCL-*b*-PMAA copolymer was synthesized by the atom transfer radical polymerization (ATRP) of *tert*-butyl methacrylate from a bifunctional PCL macroinitiator, followed by hydrolysis of the P*t*BMA blocks. The synthesis procedure involved three stages, as shown in Figure 1. Firstly, PCL-based bifunctional macroinitiator (Br-PCL-Br) was obtained by esterification of the hydroxyl groups of PCL-diol with bromoisobutyryl bromide in the presence of TEA at 0 °C. Next, *t*BMA was polymerized from Br-PCL-Br in the presence of a Cu/PMDETA complex as a catalyst system in anisole at 70 °C, via the ATRP mechanism. The final step was the conversion of the *tert*-butyl groups to carboxylic groups by selective hydrolysis with TFA.

**Figure 2:**
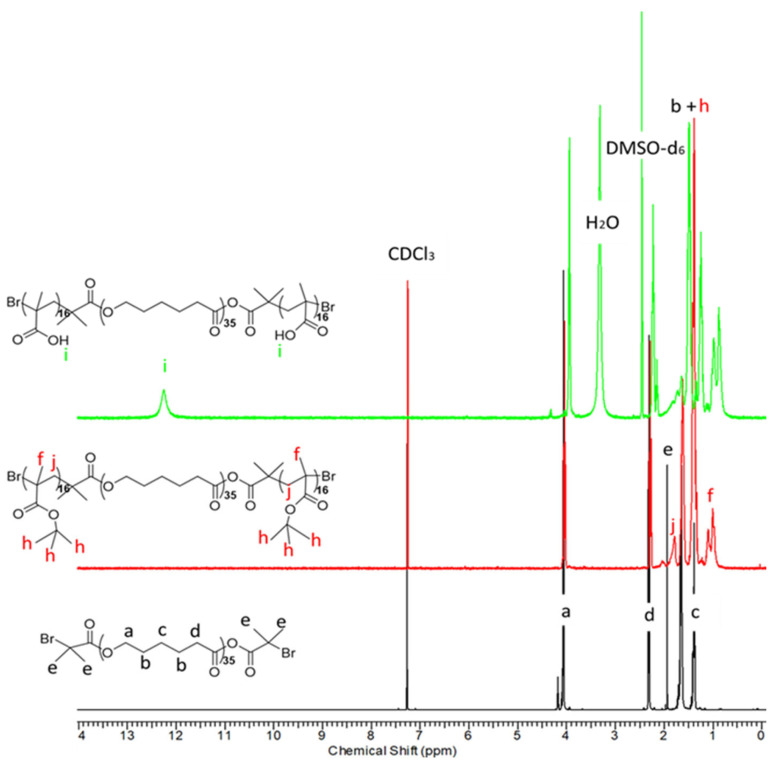
^1^H-NMR spectra of PCL-based ATRP initiator (bottom), poly(*t*-butyl methacrylate)_16_-*b*-poly(ε-caprolactone)-*b*-poly(*t*-butyl methacrylate)_16_ precursor (middle) in CDCl_3_, and poly(methacrylic acid)_16_-*b*-poly(ε-caprolactone)_35_-*b*-poly(methacrylic acid)_16_ amphiphilic triblock copolymer (top) in DMSO-d_6_.

The chemical structure of the precursors, Br-PCL-Br and P*t*BMA-*b*-PCL-*b*-P*t*BMA, and the amphiphilic triblock copolymer, PMAA-*b*-PCL-*b*-PMAA, were confirmed by ^1^H-NMR spectroscopy (Figure 2). Expectedly, very high degree of esterification of PCL (ca. 100%) was calculated from the peak integral ratio of the methylene protons of PCL (-CH_2_, δ = 4.06 ppm, a) and methyl protons (-CH_3_, δ = 1.93 ppm, e) of bromoisobutyryl bromide group. The polymerization of *t*BMA afforded new intense peak at δ = 1.42 ppm, corresponding to the methyl protons of the *tert*-butyl group (h) and a less intense signals from the methylene (-CH_2_, in the interval δ = 2.3–1.8 ppm, j) and methyl protons (-CH_3_, in the interval δ = 1.8–1.03 ppm, f) of the P*t*BMA segments in NMR spectrum of the collected reaction product. This result revealed that a P*t*BMA-*b*-PCL-*b*-P*t*MBA triblock copolymer was successfully synthesized, while the calculated average degree of polymerization (DP) of P*t*BMA was 32 (2 × 16 units). The conversion of P*t*BMA to PMAA was confirmed from the loss of the characteristic peak of *tert*-butyl protons at 1.42 ppm (h) and the presence of new proton signals, assigned to -COOH protons of PMAA blocks at δ = 12.26 ppm (Figure 2, top). It should be mentioned that the hydrolysis with TFA, performed at a mild condition, did not affect the ester bonds of PCL.

**Figure 3:**
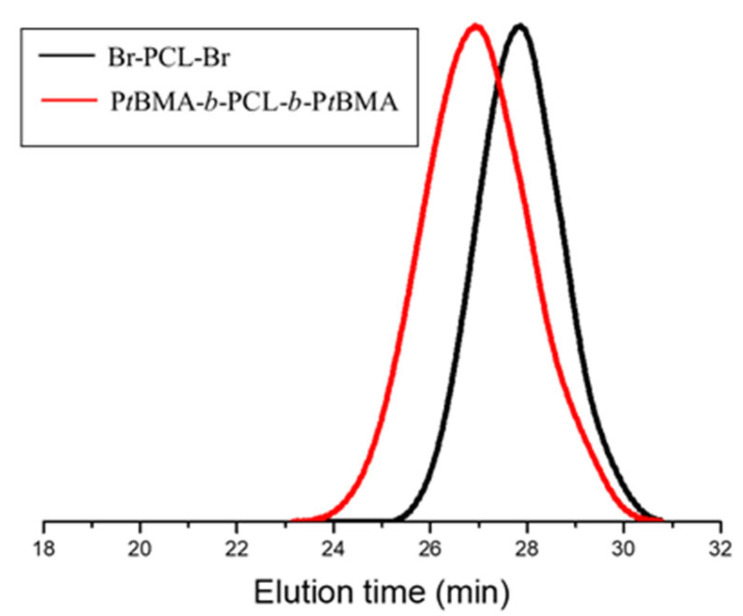
GPC chromatograms of Br-PCL-Br and P*t*BMA_16_-*b*-PCL_35_-*b*-P*t*BMA_16_ with THF as the eluent.

GPC chromatograms of the macroinitiator and P*t*BMA-*b*-PCL-*b*-P*t*BMA precursor are shown in Figure 3. The two polymers exhibited monomodal curves with relatively narrow molar mass distribution. This fact indicated a high efficiency of the Br-PCL-Br initiator and good control over the ATRP process. The number-average molar masses and dispersity indices of the precursors, calculated from the GPC analysis, are provided in Table 1.

### 3.2. Preparation and Characterization of Polymeric Micelles

**Figure 4:**
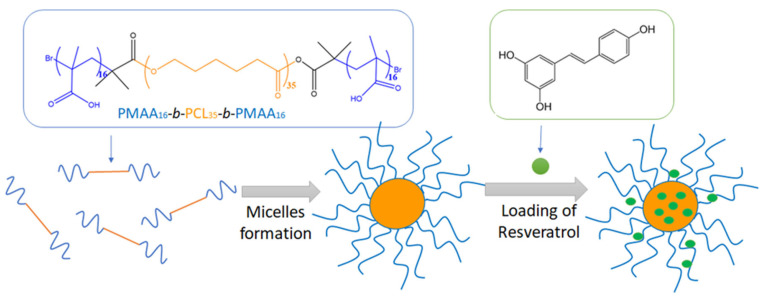
Schematic representation of the formation of resveratrol-loaded micellar nanocarriers via self-assembly of the PMAA_16_-*b*-PCL_35_-*b*-PMAA_16_ triblock copolymer in water.

The micellar nanocarriers were prepared by self-assembly of PMAA_16_-*b*-PCL_35_-*b*-PMAA_16_ in water (Figure 4). The copolymer was not directly soluble in water, and therefore it was first dissolved in an acetone/THF mixture and then added dropwise to deionized water. Next, the organic solvents were evaporated to afford a transparent aqueous colloid (1 gL^−1^). In water, the hydrophobic PCL blocks of the copolymers underwent phase segregation and formed the hydrophobic core of the micelles, whereas the hydrated PMAA chains built the corona of the micelles. The role of PCL was not only to solubilize the water insoluble resveratrol, but also to impart biodegradability of the carrier. Moreover, the semi-crystalline nature of PCL was expected to provide a superior structural stability of the carriers (frozen micelles) and to prevent a possible micelle disaggregation below the critical micelle concentration (CMC) [36].

**Figure 5:**
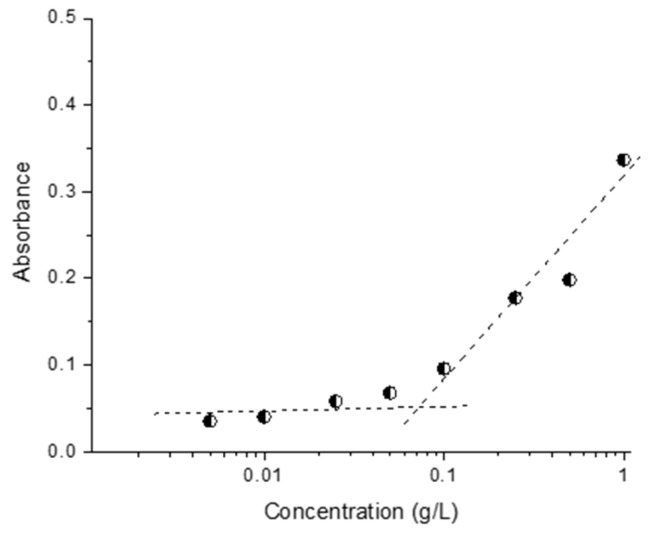
Determination of the critical micelle concentration of PMAA_16_-*b*-PCL_35_-*b*-PMAA_16_ triblock copolymer in water.

**Figure 6:**
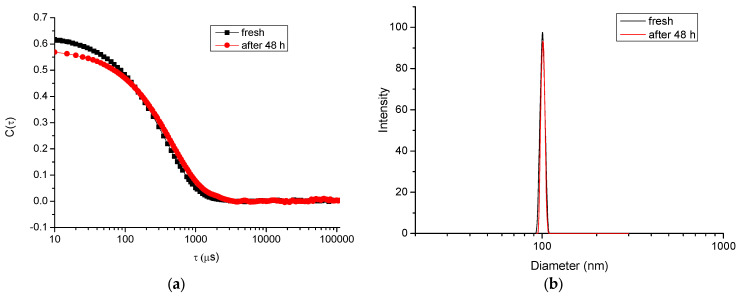
Autocorrelation function (**a**) and hydrodynamic diameter distribution (**b**) plots of PMAA_16_-*b*-PCL_35_-*b*-PMAA_16_ micelles in water.

Firstly, the main physicochemical characteristics of blank micelles was investigated. The CMC, as determined by the dye (DPH) solubilizing method, was 0.076 gL^−1^ (Figure 5). This method is commonly used for self-assembling copolymer systems and provides reliable results; in an aqueous polymer solution, the hydrophobic dye DPH has minimal UV absorbance, while in the hydrophobic environment of the micellar core, the intensity of the DPH spectrum, with a maximum at 356 nm, is much more pronounced [37]. Taking into account the CMC of the copolymer, the micelles intended for the loading of resveratrol were prepared at a concentration of 1 gL^−1^, which is one order of magnitude higher than the CMC value. DLS measurements of freshly prepared blank micelles revealed a monomodal particle size distribution, with a mean hydrodynamic diameter (D_h_) of 102 nm (Figure 6). Next, the colloid stability of micelles after 48 h storage at room temperature (25 °C) was monitored. The autocorrelation function and size-distribution plots did not undergo significant changes, indicating that, at these conditions, the micelles do not tend to agglomerate/precipitate (Figure 6).

**Figure 7:**
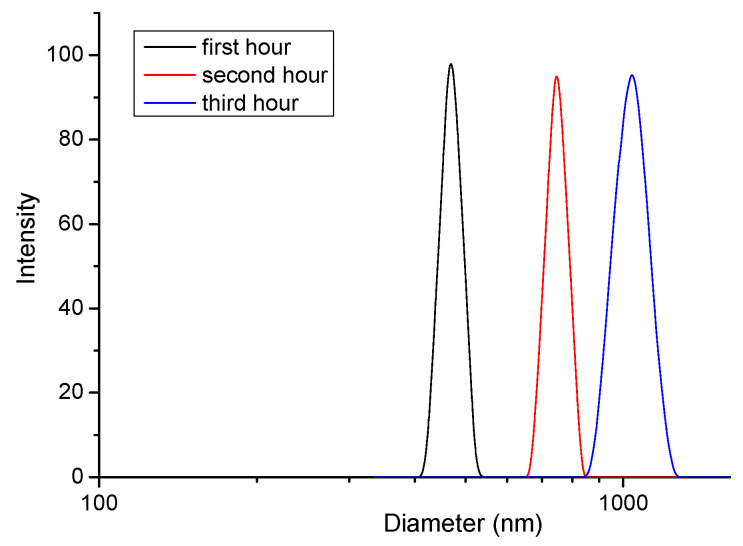
Hydrodynamic diameter distribution of PMAA_16_-*b*-PCL_35_-*b*-PMAA_16_ micelles in acidic media (pH 1.2) at different time.

The behavior of micelles in acidic media (resembling the stomach conditions) was completely different. Once placed in a buffer solution of pH 1.2, the micelles started to aggregate, and within the time-interval of 3 h, some precipitate was observed. DLS data also confirmed the aggregation process, since particles much larger than the initially formed micelles were found in the samples analyzed after 1, 2 and 3 h, respectively (Figure 7).

**Figure 8:**
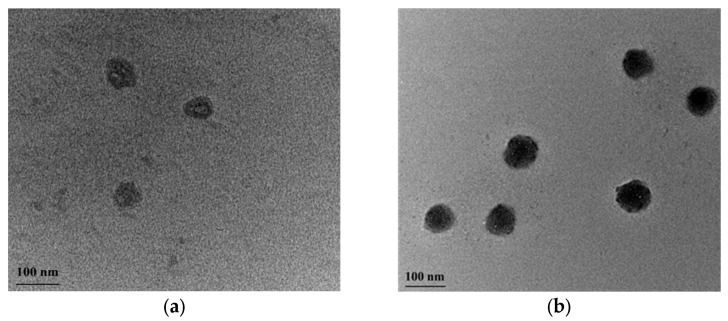
Selected transmission electron micrographs of freshly prepared (**a**) and stored at pH 1.2 for 2 h (**b**) PMAA_16_-*b*-PCL_35_-*b*-PMAA_16_ micelles. The micelles on the right micrograph contain DPH.

Most probably, protonation of PMAA segments at low pH favored the formation of hydrogen bonds between carboxyl groups, leading to reduced repulsion of PMMA coronas. Therefore, the colloid stability of the system was lost. It was also important to know if the PCL core of micelles degraded in acidic media for the relatively short time of exposure (2–3 h). Degradation of the micellar core will cause disintegration of the carrier, which is considered disadvantageous at that stage of drug delivering. To obtain an idea about this, PMAA_16_-*b*-PCL_35_-*b*-PMAA_16_ micelles were loaded with DPH, the pH of media was adjusted to 1.2 and the system was stored for 3 h. Analysis with UV-vis spectroscopy revealed existence of intense spectrum with a maximum at 356 nm, meaning that the hydrophobic micellar cores were not destroyed. In addition, TEM analyses of blank micelles, before and after storage in medium with pH 1.2, confirmed that the spherical morphology and the nanoscopic dimensions of the carriers were preserved (Figure 8). After passing the stomach, the nanocarriers enter the small intestine and therefore their behavior in an environment with pH = 6.8 was investigated as well. DLS results (Table 2) showed that the system is colloidally stable, and the D_h_ and ƺ-potential of carriers are identical with the characteristics of micelles in pure water.

### 3.3. Drug-Loaded Nanocarriers

**Figure 9:**
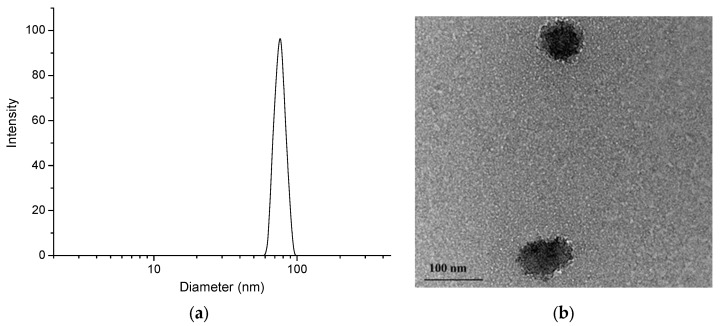
Hydrodynamic diameter distribution plot (**a**) and TEM micrograph (**b**) of resveratrol-loaded PMAA_16_-*b*-PCL_35_-*b*-PMAA_16_ micelles.

The loading of the hydrophobic polyphenol resveratrol into the preformed nanocarriers was conducted via the solvent evaporation method in a mass ratio between the polymer and the active substance of 15:1 (Figure 4). An encapsulation efficiency of 72% and a loading degree of 0.067 mg/mL were achieved. Notably, the affinity of the hydrophobic resveratrol to the core of the micelles was not very pronounced, which leads to not so high encapsulation efficiency and loading capacity. Moreover, the hydrodynamic diameter of resveratrol-loaded micelles was decreased compared to the blank micelles, which is probably due to shrinkage of PMMA corona because of interactions between the polymer and the drug (Figure 9a). Such interaction was also indicated by the slight increase of the ƺ-potential value (Table 2). On the other hand, the contrast of the micelles observed by TEM (Figure 9b) was increased. Based on these results, we can assume that some of the drug molecules are embedded in the micellar corona rather than the core.

**Figure 10:**
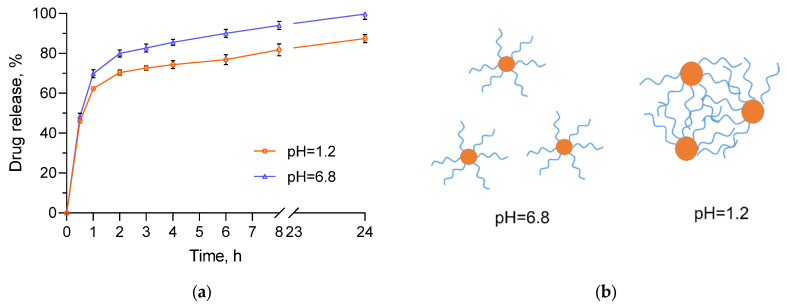
In vitro release tests of resveratrol-loaded micelles in media with pH = 1.2 and pH = 6.8 (**a**) and schematic presentation of micelles in both media (**b**).

The in vitro release tests of resveratrol from the micelles were conducted in buffer media, which represent the pH along the gastrointestinal tract (pH of 1.2 and 6.8). As shown from Figure 10a, there was a burst effect in the beginning, followed by a sustained release. The rapid release in the first one hour confirmed our hypothesis for partial location of resveratrol in the PMAA corona of aggregates. Two processes may interfere the incorporation of resveratrol into the interior of PCL core: (i) hydrogen bonding between PMMA (-COOH groups) and drug molecules (-OH groups); and (ii) crystallization of PCL, leading to the formation of domains in the core with a higher structural order [38], in which the penetration of drug molecules is hard. Concerning different media, the release of resveratrol in the medium with pH of 6.8 was slightly faster than the release in the medium with pH of 1.2. For instance, at the fourth hour, approximately 86% of resveratrol was released in the medium resembling the intestines’ pH (6.8), while at the gastric pH (1.2), the released drug was 74%. It is important to note that the solubility of the pure drug in these media is almost equal [39,40]. Thus, the different release rate was related to the properties of the polymer carrier in both media. In particular, the slower release in the acid medium could be due to the protonation of the carboxylic groups of PMMA (pKa 4.65) and subsequent aggregation of the micelles. In this case, the diffusion of resveratrol embedded into the aggregates was hindered to some extent by the formed physical network of PMMA chains (Figure 10b). This phenomenon was confirmed by kinetic analysis of the release data (Table 3). The results for the release in the acidic medium are correlated most closely with the Higuchi model (highest regression coefficient), which proved that the release was controlled by diffusion. Interestingly, the kinetic analysis of the data obtained for the phosphate buffer (pH 6.8) revealed that the release followed first-order process rather than Higuchi model. In this case, since there was no aggregation, the release depended mainly on the remaining drug concentration in the micelles. Nevertheless, the release process in both media ensured 100% delivery of resveratrol, which is advantageous when compared to the incomplete dissolution the pure drug (approximately 10% for 24 h). Thus, from a biopharmaceutical point of view, the developed micellar system was considered more appropriate for oral administration than the pure resveratrol. Importantly, the improved biopharmaceutical properties of this poorly soluble drug could further increase its bioavailability, which was very low [30].

**Figure 11:**
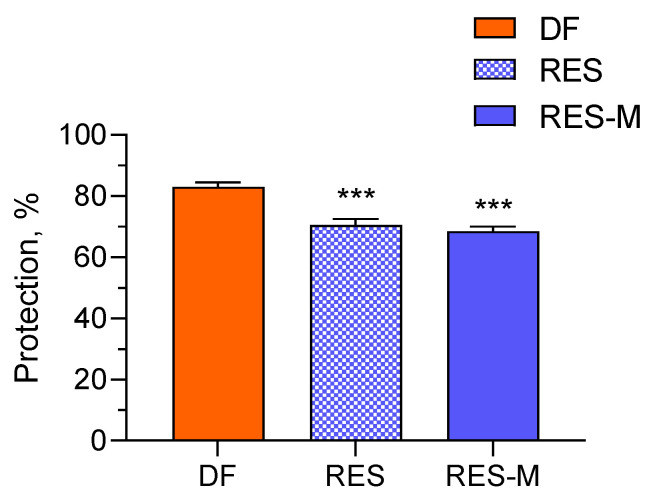
In vitro inhibition of denaturation of albumin via treatment with diclofenac sodium (DF), pure resveratrol (RES) and resveratrol loaded into the micelles (RES-M). *** *p* < 0.001 vs. diclofenac control group.

Compounds with antioxidant activity, such as resveratrol, could capture reactive oxygen species and free radicals that are produced during inflammatory process [41]. In this view, our next task was to evaluate in vitro the anti-inflammatory activity of pure and micellar resveratrol via the albumin denaturation assay. More specifically, the denaturation of albumin provokes the formation of autoantigens that initiate type III hypersensitive reaction, leading to inflammation [42]. Thus, the potential of pure and encapsulated resveratrol to inhibit a heat-induced albumin denaturation was examined and compared. Diclofenac sodium, a standard anti-inflammatory drug, was applied as a positive control. As shown in Figure 11, the incubation of diclofenac with albumin led to approximately 83% inhibition of denaturation. Similarly, a strong inhibition of albumin denaturation with this drug was observed from other study groups [34,43]. The pure and micellar resveratrol also showed sufficient potential to inhibit albumin denaturation although their effect was lower than this of diclofenac, in particular 70.5% and 68.5%, respectively. These results indicated that both forms of resveratrol (pure and micellar) could be considered effective anti-inflammatory candidates. It is important to note that the inclusion of resveratrol into the micelles did not decrease its effect (no statistical difference between the groups).

**Figure 12:**
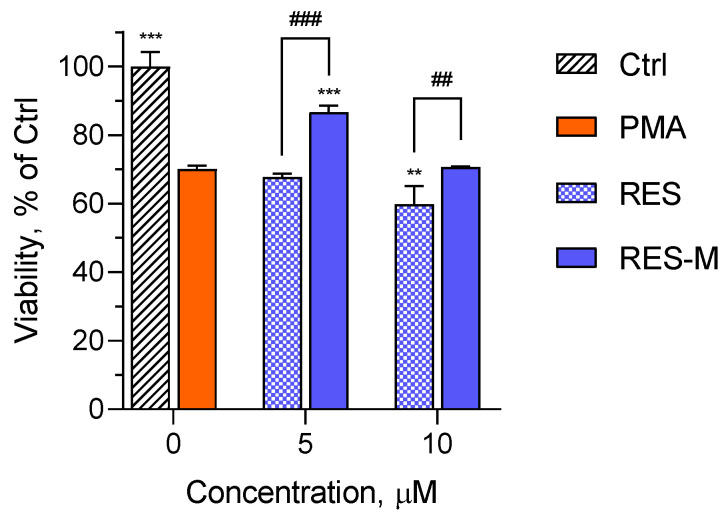
Viability of damaged HT-29 cells upon pre-treatment with pure resveratrol (RES) and micellar resveratrol (RES-M). Abbreviations: Ctrl—non-damaged HT-29 cells; PMA—HT-29 cells damaged with PMA-differentiated EOL-1 cells. *** *p* < 0.001; ** *p* < 0.01 vs. PMA-treated group; ### *p* < 0.001; ## *p* < 0.01 between cells treated with pure and micellar resveratrol.

To prove the anti-inflammatory activity of resveratrol, its protective effect against the damage of mucosa epithelial HT-29 cells was evaluated. The method included pre-treatment of HT-29 cells with pure or micellar resveratrol followed by cytotoxic damage via treatment with differentiated eosinophilic EOL-1 cells. Differentiation of EOL-1 cells and their functional activation, e.g., production of inflammatory cytokines, was induced by incubation with phorbol 12-myristate 13-acetate (PMA) [44]. The co-incubation of PMA-treated EOL-1 cells with HT-29 cells resulted in a 30% decrease in the viability of HT-29 cells (Figure 12). It was found out that the pre-treatment of HT-29 cells with the empty micelles did not provide protective effect. However, there was a significant difference between the effects after pre-treatment with pure and micellar resveratrol. At the lower concentration (5 µM), there was protection only when the cells were pre-treated with the micellar resveratrol. In this case, the micellar resveratrol resisted to inflammatory cytokines and maintained 90% viability in HT-29 cells. On the contrary, the pre-treatment with pure resveratrol did not provide protection at all. Furthermore, with increasing the concentration (10 µM), the pure resveratrol revealed a tendency to exert an additional cytotoxic effect. In fact, this observation correlated with previous studies which reported that increasing the concentration of resveratrol above a particular level could lead to occurrence of prooxidant and toxic effects [45,46]. For comparison, the micellar resveratrol had no protective effect, but did not exert cytotoxicity. As a conclusion, the loading of resveratrol into the micelles improved its protective effect in the developed method. The results are in agreement with other studies that have reported an improvement of antioxidant and anti-inflammatory activity of resveratrol upon its formulation in nanoparticles [47,48,49,50].

## 4. Conclusions

A new system for oral delivery of the hydrophobic polyphenol resveratrol, based on PMAA-*b*-PCL-*b*-PMAA triblock copolymer micelles, was developed. The formulated micelles possessed favorable characteristics such as nanoscale size, monomodal size distribution, negative surface charge and superior structural stability. The micelles readily encapsulated resveratrol and thereby significantly increased its solubility in aqueous media, while providing complete drug release. This behavior demonstrated the potential of the developed micelles to overcome the main biopharmaceutical problem of the pure drug. Furthermore, the micellar resveratrol protected epithelial cells against the damage provoked by inflammatory cytokines. For comparison, the pure drug did not protect the cells at all. These results revealed an opportunity to achieve a better anti-inflammatory effect of resveratrol in treating inflammatory gastrointestinal diseases by using the PMAA-*b*-PCL-*b*-PMAA-based micellar formulation of the drug.

## Figures and Tables

**Table 1:** Composition and molecular characteristics of Br-PCL-Br, P*t*BMA-*b*-PCL-*b*-P*t*BMA and PMAA-*b*-PCL-*b*-PMAA.

Sample Code	M_n_^NMR^ (g mol^−1^)	M_n_^GPC^ (g mol^−1^)	M_w_/M_n_
Br-PCL_35_-Br	4400	5000	1.24
P*t*BMA_16_-*b*-PCL_35_-*b*-P*t*BMA_16_	8500	10,400	1.27
PMAA_16_-*b*-PCL_35_-*b*-PMAA_16_	6700	-	-

**Table 2:** Physicochemical parameters of PMAA_16_-*b*-PCL_35_-*b*-PMAA_16_ block copolymer micelles in different media.

Sample	D_h_ (nm)	Zeta-Potential (mV)
Blank micelles in water	102 ± 3	−30 ± 4
Blank micelles in buffer (pH 1.2, 1 h)	745 ± 10	-
Blank micelles in buffer (pH 6.8)	103 ± 3	−16 ± 3
Drug-loaded micelles in water	78 ± 2	−24 ± 3

**Table 3:** Correlation coefficients (r^2^) for zero-order, first-order and Higuchi model calculated through kinetic analysis of in vitro release data (2–24 h).

pH of the Medium	Zero-Order	First-Order	Higuchi Model
pH 1.2	0.8487	0.9177	0.9325
pH 6.8	0.6828	0.8645	0.8107

## Data Availability

Not applicable.
